# Reduction of friction and wear by grooves applied on the nanoscale polished surface in boundary lubrication conditions

**DOI:** 10.1186/1556-276X-9-226

**Published:** 2014-05-08

**Authors:** Alexander U Stelmakh, Yuriy V Pilgun, Sergiy O Kolenov, Alexey V Kushchev

**Affiliations:** 1 National Aviation University, Kyiv, 03058, Ukraine; 2 Taras Shevchenko National University of Kyiv, Kyiv, 01601, Ukraine

**Keywords:** Friction force, Wear reduction, Microstructured surface, Compressive-vacuum, Boundary lubrication, Sliding tribosystem

## Abstract

The evolution of a friction surface geometry with initially directed microscale grooves on a nanoscale polished surface in ring-on-block sliding contact is studied experimentally. Reduced wear and friction is observed when the orientation of grooves coincides with the direction of sliding. A new compressive-vacuum hypothesis of friction force nature under a condition of boundary lubrication is proposed, which successfully explains the observed phenomena. Grooves supply lubricant into the contact zone and facilitate its devacuumization, which lead to substantial reduction of surface wear. The obtained results enable developing optimized roughness profiles of friction surfaces to create high-performance durable friction units.

## Background

Modern tribology has a considerable amount of experimental data about a friction process under conditions of boundary lubrication. Such process is always accompanied by a wear, which usually is associated with adhesion of sliding bodies [1]. According to current theories of friction and wear [1-3], friction force *F*_fr_ can be separated into two basic components: mechanical deformation component *F*_def_ and adhesive component *F*_adg_

(1) $$F_{\mathrm{fr}}=F_{\mathrm{def}}+F_{\mathrm{adg}}.$$

Deformation component is associated with local elastic deformation of solids under conditions of elastohydrodynamic lubrication, while adhesive component can be considered as a worsening factor appearing when direct contact of bodies become inevitable due to lubricant film failure. In typical situations, adhesive force is many orders of magnitude larger than the deformation component, so most research work is concentrated on the search for methods of minimizing adhesive component of the friction force. Nevertheless, treatment of a friction process as a mixture of elastohydrodynamic and boundary lubrication regime is not complete. It is usually assumed that for elastohydrodynamic lubrication regime hydraulic pressure of lubricant equals to contact stresses [1-3], which might not be the case in reality. The main condition for elastohydrodynamic regime is continuity of lubricant during flow over contact, but this condition is not satisfied in many experiments because cavitation at the contact exit is quite a common effect [1,4,5]. Cavitation is the result of the so-called negative pressure conditions, when liquid pressure becomes much lower than the atmospheric value, and fast decompression releases stored gases. The occurrence of cavitation is a direct evidence that hydraulic pressure in the contact zone is not necessarily higher than the pressure in the outside regions, but instead could be much lower than the external pressure. Suction produced by lowered pressure put additional strain on sliding bodies and causes adverse effect on friction because it pulls surfaces towards each other. We believe that such decompressive mechanism of friction really happens in practice and should be considered along with deformation and adhesive force components. Thus, current theory of friction should be extended and include force components associated with decompression to match experimental data.

**Figure 1:**
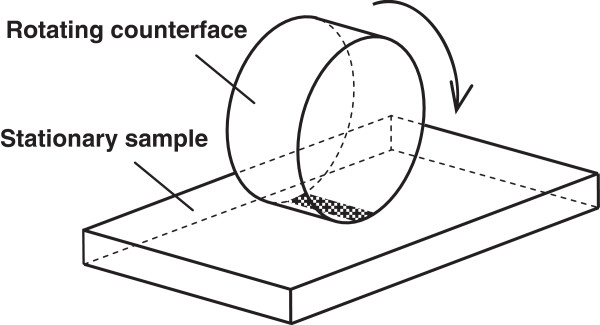
A sliding tribosystem model: cylindrical roller rotating over the motionless block.

**Figure 2:**
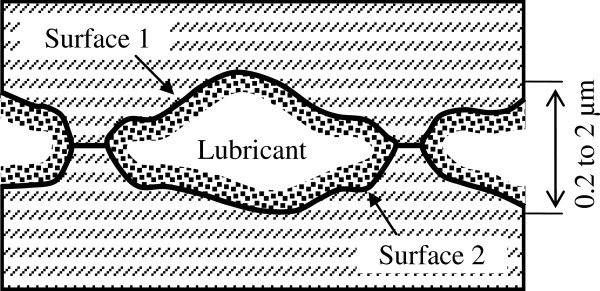
Closed volumes formed by valleys between peaks on contacting surfaces.

Load-carrying capacity of lubricants at extreme pressure conditions is routinely studied in the Timken test ring-on-block configuration [6] (Figure 1). This geometry proved to be useful for modeling sliding bearing systems. Our compressive-vacuum hypothesis of friction for such configuration is discussed as follows: When two rough surfaces are pressed together, the initial contact occurs between peaks of the roughness. These peaks are deformed under compression forces and form ‘contact spots.’ Isolated valleys with lubricant are formed between the compressed peaks forming closed contour lines (Figure 2). During the entry phase, the pressure of lubricant in such closed valleys increases. As a result, the lubricant is squeezed out into nearby valleys with smaller pressure. Compression of the peaks continues until the maximum contact stress is reached. After that, when valleys approach the exit of the contact region, the contact stress decreases and a vacuumization process in closed valleys begins. Separation of surfaces during rolling acts as an external force which forcibly increases the volume of the closed valleys. As a result, pressure in the closed volume of valleys is decreased and can become lower than the atmospheric pressure (thus, we use the term ‘vacuumization’). Decrease of lubricant pressure at the contact exit has twofold consequences. Firstly, friction force is substantially increased by suction produced by regions with lowered pressure. Secondly, vacuumization could lead to cavitation, which cause desorption of lubricant and expose naked surface further increasing adhesive friction. We believe such compressive-vacuum component of friction force do in fact exist in practice. We have called this component as compressive-vacuum friction force (*F*_cv_). This additional force consists of compressive component arising at the entry of the contact and a vacuum one acting on the contact exit. Therefore, Equation 1 should be rewritten as

(2) $$F_{\mathrm{fr}}=F_{\mathrm{def}}+F_{\mathrm{adg}}+F_{\mathrm{cv}}.$$

Vacuumization processes not only add to friction force but also increase wear, because produced suction forces along with contact of the naked surface make easier to damage sliding surfaces. In our opinion, wear of sliding contact could be greatly reduced by searching some methods to reduce friction force. These methods may include formation of micro-roughness of special shape on the surface. Similar approach was successfully used in [7] to reduce friction force in point-contact friction system. Though we use linear contact which differs significantly in properties, specially formed surface can also be used to reduce friction and wear. According to our compressive-vacuum hypothesis of friction, this can be done by preventing vacuumization. This idea is supported by the experimental data obtained during the friction testing of steel surfaces with specially designed micro-roughness [8,9].

## Methods

In the present work, the Timken test [6] is chosen as a physical model of a sliding tribosystem. This model corresponds to a rotating shaft on plane bearing system, which is the most widespread and also the most often friction-damaged unit in engineering.

Boundary lubrication is accompanied by wear, so additional care should be taken in experiments. It is important not to allow wear debris to cause micro-cutting damage of the contact zone on the one hand and not to allow formation of simple elastohydrodynamic (contactless) friction on the other hand. In used experimental system, the evolution of wear scar in time is controlled by microscopy, so these precautions are easily satisfied.

**Figure 3:**
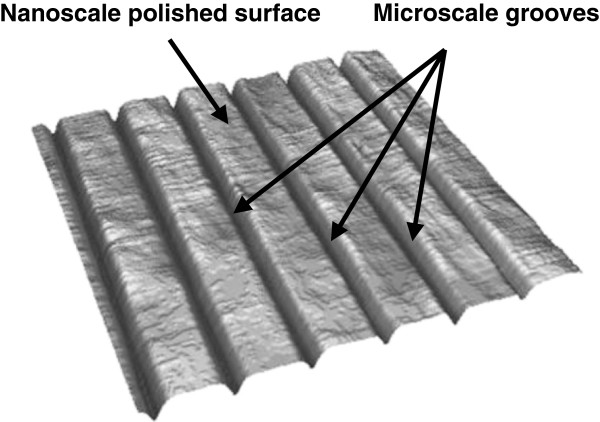
Special surface structure consisting of parallel grooves proposed for wear reduction.

On the basis of the compressive-vacuum hypothesis described above, we suppose that it is necessary to create special initial three-dimensional (3D) geometry of a sample's surface roughness which will allow to reduce compressive and vacuum hydrodynamic components of friction force and as a consequence will also reduce contribution of adhesive interaction of surfaces. For this purpose, creation of test samples with specific channels on the surface is suggested. These channels would provide bypass for the lubricant from areas entering the contact to areas leaving the contact, so reduction of vacuum in the exit region becomes possible. Such channels on a surface of test objects can be formed as parallel grooves, like shown in Figure 3. If grooves are oriented along the sliding direction, they might supply the lubricant into a vacuum zone and provide devacuumization of the compression areas of tribological system. We suppose that the formation of such directed microstructure on a surface of samples will create conditions when closed vacuum valleys in the contact zone either will not be formed at all or will be easily and quickly devacuumized. As a result, it should lead to substantial reduction friction force and surface wear.

### Experimental study

Ball-bearing steel grade ShH15 (according to the standard GOST 801-78) produced by electroslag remelting has been chosen as a material for fabrication of samples. It has international analogues: American AISI Type E52100, UNS G52986, European 100Сr6, and Japanese JIS SUJ2. This high-carbon chromium steel features high hardness, high mechanical strength, and dimensional stability. Tribological tests were carried out on the friction machine with a fixed flat-surface sample and a rotating cylindrical counterface sample. The oil IMP-10 was used as a lubricant.

A special technique for forming grooves on a sample surface with specified 3D geometry was developed. Initially, the surface of the sample was polished to a level of roughness with Ra about 0.02 μm. Then, diamond paste with size of a grain corresponding to the desired depth of grooves was applied. Movement of a polishing plane with diamond paste was performed only in one direction. Polishing with the paste actually led to controllable scratching of the surface. Polishing movements were repeated only a few times to preserve the initial nano-topography of the surface between grooves. Intermediate results were checked by the laser differential phase profilometer [10] and scanning electron microscope. As a result, ten flat samples with directional grooves had been fabricated. The depth of grooves was varied in the range from 0.3 to 2.6 μm. Rotating cylindrical counterface had no grooves on it, and surface roughness was the same as the initial roughness of samples Ra = 0.02 μm.

A multistage testing technique which mimics operation conditions of real friction units was developed. The testing procedure of each sample included the following: (1) three initial run-in stages, in which the formation of secondary structures on friction surfaces occurred; (2) the final test stage, during which tribological and rheological characteristics of a friction samples and lubricant were estimated. Each of the initial three stages was run until a length of friction equals *L* = 500 m. The final measurement stage had a length of friction *L* = 3,000 m. Ambient temperature was 20°С. Axial load 1,250 N was big enough to maintain permanent wear but not to allow plastic deformation of material. According to the Hertzian theory for considered sliding bearing [11], maximal contact load is more than 1,000 MPa (estimated not taking into account the surface roughness). The contact was fully immersed in oil, and the sliding velocity of roller over the sample with nanogeometric roughness was 0.3 m/s. For such contact load and speed, boundary lubrication regime is realized [5]. This leads to inevitable adhesive contact wear for the samples with flat surface [12].

Both samples with flat surface and with pre-formed grooves were tested. For samples with directed structure, the orientation of grooves was parallel to the direction of sliding.

## Results and discussion

**Figure 4:**
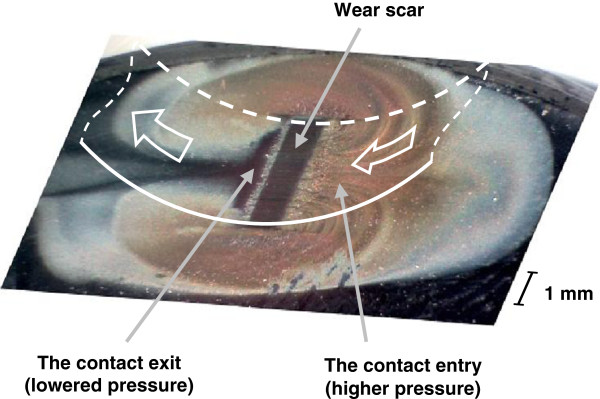
Wear scar and wear products on the surface of test sample with initially flat surface.

A typical resulting wear scar after friction test of sample with polished surface is shown in Figure 4. Wear products are seen around the contact as brown waste material. The presence of debris on the sample confirms that adhesive friction conditions are realized in the experiment and actual wear process takes place. It should be noted that wear products are gathered mostly in front of the contact entry. There are also seen two curled streams which carry away wear products around the contact from the sides. Considering that the hydrodynamic pressure in front of the contact is larger than behind it, such arrangement seems explainable. Obviously, wear products cannot be streamed directly through the contact, because the gap between sliding surfaces is very small, especially in boundary lubrication conditions. Possibly, some reverse circulating current of lubricant is formed near the contact entry, which could lead to the observed pattern of wear product deposition, but this question needs further investigations.

Fundamentally different picture is observed when the sample has grooves on the initial surface. After initial run-in stages, wear products do not accumulate anymore around the contact in substantial quantities and cannot be detected visually.

**Figure 5:**
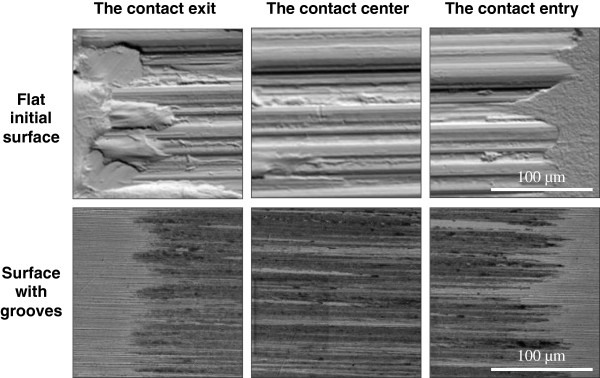
Details of wear scar after friction test for samples with flat and grooved surfaces.

Microphotographs of wear scar obtained with scanning electron microscope (SEM) show completely different topography of the surface for the case of flat and grooved samples (see Figure 5). The scar on initially flat sample reveals complex profile. It contains multiple scratches, significant number of craters, and lumps of pulled out metal, which are the result of adhesive transfer of material. Most damaged areas are located at the contact exit. Similar effect was observed earlier [13]. We think this effect is caused by vacuumization, which is strongest at the contact exit. Thus, we conclude that vacuumization is responsible for most of the adhesive wear and leads to damage of the area near the contact exit.

In the case of grooved sample, the scar has much smoother profile without any signs of adhesive interaction of surfaces. These experimental results support our assumption that grooves on the surface create bypass channels for equalization of hydrodynamic pressure in the contact region and thus prevent direct contact and adhesion of friction surfaces.

**Figure 6:**
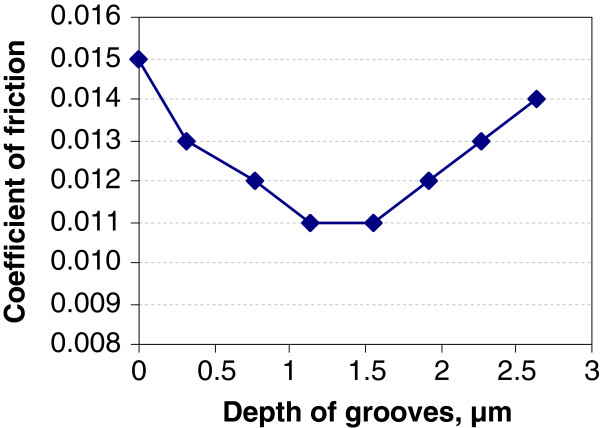
Dependence of friction coefficient on depth of grooves during final test stage.

The friction coefficient for samples with flat initial surface was about 0.015. The measured coefficient of friction for grooved samples is a little lower (see Figure 6). Dependence on groove depth is rather weak and has a minimum value 0.011 at a groove depth around 1.3 μm. It can be a sign of more advantageous conditions in the friction contact provided by grooves. With increasing depth of grooves, coefficient of friction increases. It can be explained that for bigger grooves relative area of nanoscale polished base surface is reduced, which has negative effect on friction due to plastic deformation of material.

Experimental findings may look unexpected, because usually highly polished surface has better friction performance than the rough one. In our case, flat surface with roughness parameter Ra = 0.02 μm has high wear rate in boundary lubrication, while samples with much more coarse (0.3 to 2.6 μm), but directed variations of surface profile, demonstrate almost no wear. The positive effect is obviously based on proper orientation of grooves. When grooves are oriented not along the sliding direction, but perpendicular to it, friction coefficient becomes much larger: 0.05 to 0.08. Conceivably, improper orientation does not provide channels needed for devacuumization of the exit region and also cause adverse effect on friction because linear contact can ‘fall down’ into some of grooves which increase contact stresses. Also, important role plays initial finishing of the surface between grooves, which should be of nanometer scale.

## Conclusions

In the course of tribological tests of cylindrical roller sliding over a rough surface, a phenomenon of the friction and wear reduction is observed in the case when specially oriented grooves are applied to the surface of the sample. The proposed compressive-vacuum theory explains this phenomenon by devacuumization of the contact exit area. Grooves oriented along the sliding direction provide channels needed to equalize hydrodynamic pressure in the contact area, which helps avoid the formation of region with lowered pressure and decreases a probability of adhesive interaction of the surfaces. Effectiveness of this process depends on the depth of grooves.

The proposed theory can give important insight into the true nature of processes leading to adhesive contact of friction surfaces in boundary lubrication conditions. It is proposed to include compressive-vacuum component of friction force into consideration, as lowered pressure can create substantial resistance to movement due to suction effects. Considered effects are of great practical significance, because technologically simple preparation of friction surfaces can greatly reduce wear in tribosystems.

## Competing interests

The authors declare that they have no competing interests.

## Authors' contributions

AUS is the author of the original compression-vacuum hypothesis of friction, proposed the ideas for the experiments, carried out the general coordination of the work, participated in performing the experiments, and analyzed the obtained results drawing conclusions. YVP drafted the manuscript and participated in the mathematical processing and analysis of data obtained from laser differential phase profilometer. SOK obtained the experimental data for wear scars with laser differential phase profilometer and participated in plotting and analyzing data. AVK performed the tribological tests, obtained pictures of wear scars with scanning electron microscope, and participated in analyzing data. All authors read and approved the final manuscript.
